# Structural and functional neural adaptations in obstructive sleep apnea: An activation likelihood estimation meta-analysis

**DOI:** 10.1016/j.neubiorev.2016.03.026

**Published:** 2016-06

**Authors:** Masoud Tahmasian, Ivana Rosenzweig, Simon B. Eickhoff, Amir A. Sepehry, Angela R. Laird, Peter T. Fox, Mary J. Morrell, Habibolah Khazaie, Claudia R. Eickhoff

**Affiliations:** a Sleep Disorders Research Center, Kermanshah University of Medical Sciences (KUMS), Kermanshah, Iran; b National Brain Mapping Center, Shahid Beheshti University (General & Medical campus), Tehran, Iran; c Sleep and Brain Plasticity Centre, Department of Neuroimaging, IOPPN, King's College and Imperial College, London, UK; d Institute of Clinical Neuroscience & Medical Psychology, Heinrich Heine University Düsseldorf, Düsseldorf, Germany; e Institute of Neuroscience and Medicine (INM-1), Research Center Jülich, Jülich, Germany; f Division of Neurology, Faculty of Medicine, University of British Columbia, Vancouver, BC, Canada; g Department of Physics, Florida International University, Miami, FL, USA; h Research Imaging Institute, University of Texas Health Science Center, San Antonio, TX, USA; i South Texas Veterans Health Care System,San Antonio, TX 78229, USA; j Academic Unit of Sleep and Breathing, National Heart and Lung Institute, Imperial College London, UK; k NIHR Respiratory Disease Biomedical Research Unit at the Royal Brompton and Harefield NHS Foundation Trust and Imperial College London, UK; l Department of Psychiatry, Psychotherapy, and Psychosomatics, RWTH Aachen University, Aachen, Germany

**Keywords:** Voxel-based morphometry (VBM), Functional magnetic resonance imaging (fMRI), Insula, Amygdala, Hippocampus, Obstructive sleep apnea

## Abstract

Obstructive sleep apnea (OSA) is a common multisystem chronic disorder. Functional and structural neuroimaging has been widely applied in patients with OSA, but these studies have often yielded diverse results. The present quantitative meta-analysis aims to identify consistent patterns of abnormal activation and grey matter loss in OSA across studies. We used PubMed to retrieve task/resting-state functional magnetic resonance imaging and voxel-based morphometry studies. Stereotactic data were extracted from fifteen studies, and subsequently tested for convergence using activation likelihood estimation. We found convergent evidence for structural atrophy and functional disturbances in the right basolateral amygdala/hippocampus and the right central insula. Functional characterization of these regions using the BrainMap database suggested associated dysfunction of emotional, sensory, and limbic processes. Assessment of task-based co-activation patterns furthermore indicated that the two regions obtained from the meta-analysis are part of a joint network comprising the anterior insula, posterior-medial frontal cortex and thalamus. Taken together, our findings highlight the role of right amygdala, hippocampus and insula in the abnormal emotional and sensory processing in OSA.

## Highlights

• The right basolateral amygdala, the hippocampus and the right insular cortex are important nodes in obstructive sleep apnea (OSA).
• Functional characterization of these regions suggested associated dysfunction of emotional, sensory, and limbic processes in OSA.
• Connectivity analysis demonstrated that these regions are part of a joint network comprising the anterior insula, posterior-medial frontal cortex and thalamus.

## 1 Introduction

Obstructive sleep apnea (OSA) is a chronic disorder that arises from recurrent partial or complete pharyngeal obstruction during sleep (Hiestand et al., 2006, Jordan et al., 2014, Kapur, 2010, Netzer et al., 2003, Rosenzweig et al., 2015). In patients with OSA, this leads to nocturnal apneas and hypopneas, intermittent hypoxia, reoxygenation and hyper-/hypocapnia events, along with sleep fragmentation, and changes in cerebral blood flow (Baril et al., 2015, Shiota et al., 2014, Yadav et al., 2013). The prevalence of OSA is noticeable in general population and around 50% in patients with cardiovascular or metabolic disorders (Khazaie et al., 2013, Khazaie et al., 2011, Lévy et al., 2015, Lurie, 2011).

Several recent studies highlighted that OSA contributes to emotional and cognitive decline, and it is increasingly considered as one of the rare modifiable risk factors for neurodegenerative dementia (Osorio et al., 2015, Rosenzweig et al., 2015, Yaffe et al., 2014, Yaffe et al., 2011). If untreated, OSA can result in varying degrees of cognitive deficits such as difficulties with attention, memory, executive functioning, and quality of life (Kryger et al., 2011, Rosenzweig et al., 2015). In addition, excessive daytime sleepiness, labile interpersonal relationships, and decreased work and school efficiency have all been documented in OSA patients (Kryger et al., 2011, Rosenzweig et al., 2015, Twigg et al., 2010). Moreover, it is recognized that OSA patients are two to thirteen times more likely to experience a driving-related traffic accident (Ellen et al., 2006, Karimi et al., 2015a, Karimi et al., 2015b, Khazaie and Maroufi, 2014). Such accidents are more likely to occur in those who manifest greater daytime sleepiness, but are not necessarily related to sleepiness alone (Ellen et al., 2006, Weaver and George, 2011). In addition to cognitive and emotional deficits, increased prevalence of OSA in several psychiatric disorders has been reported, of which major depressive disorder (MDD), anxiety, and posttraumatic stress disorder (PTSD) appear best documented (Gupta and Simpson, 2015, Sharafkhaneh et al., 2005).

It has been suggested that adaptive and maladaptive processes both occur in patients with OSA in response to hypoxemia (Rosenzweig et al., 2015). The fine balance of these processes, and its eventual impact on neurocognitive and emotional performance, will depend on the stage of this dynamic process, effects on other organ systems, cognitive reserve, and idiosyncratic susceptibility (Lavie, 2015, Rosenzweig et al., 2016a, Rosenzweig et al., 2016b, Rosenzweig et al., 2015, Sforza and Roche, 2012). Although these deficits are not always reversed with treatment (McDaid et al., 2009), a meta-analysis (Marshall et al., 2006) and a meta-review (Bucks et al., 2013) suggest beneficial effects of treatment (e.g. continuous positive airway pressure (CPAP)) on cognitive performance, sleepiness and neural injury in patients with OSA.

Over the last three decades, numerous structural and functional neuroimaging studies, including voxel-based morphometry (VBM), task functional magnetic resonance imaging (fMRI), and resting-state fMRI (rs-fMRI) have been conducted on patients with OSA. Structural and functional MRI imaging studies, however, often point to diverse results in OSA (Celle et al., 2015, Morrell and Glasser, 2011). The variability of the findings has been suggested to be due to relatively small sample sizes, with heterogeneous patient groups that differed in several key respects (e.g. diagnostic criteria, IQ, age, gender, and the imaging acquisition, preprocessing, and analysis methods); for more detailed discussion, please refer to (Gozal, 2013, Macey, 2012, Morrell and Glasser, 2011). To date, OSA structural studies have used a spatially unbiased analytical approach, such as commonly used mass-univariate approaches that rely on conservative statistical thresholds mandated by the large number of voxels compared between-groups (Ashburner and Friston, 2000). On the other hand, task fMRI studies used a variety of paradigms to study functional disturbances in particular disease. Recently, the activation likelihood estimation (ALE) method has been proposed as a useful methodology that, using coordinate-based meta-analyses (CBMA), provides a powerful tool to attain a synoptic view of distributed neuroimaging findings and different neuroimaging methods (e.g. structural and functional) in an objective and quantitative fashion (Eickhoff et al., 2009, Turkeltaub et al., 2002). More specifically, CBMA method searches for “where” in the brain the amount of convergence between reported foci is more than expected by chance, which yields to statistical inference on the integration of previous findings (Eickhoff and Bzdok, 2013, Eickhoff et al., 2012, Laird et al., 2009a, Turkeltaub et al., 2002).

Only structural OSA studies were hitherto analysed using the ALE method (Weng et al., 2014), and in order to fully address some of the previously raised concerns in the field about the diversity of these findings, we undertook the ALE meta-analysis of both functional and structural abnormalities recorded in patients with OSA. Our aim was to elucidate converging findings and to emphasize important brain nodes as highlighted via different neuroimaging modalities. We then functionally characterized the obtained regions that showed neurobiological aberrations in OSA patients by means of the BrainMap database, and also assessed their brain wide co-activation patterns to reveal networks that are (conjointly) connected to these obtained areas.

## 2 Methods

### 2.1 Search strategies and study selection

**Fig. 1:**
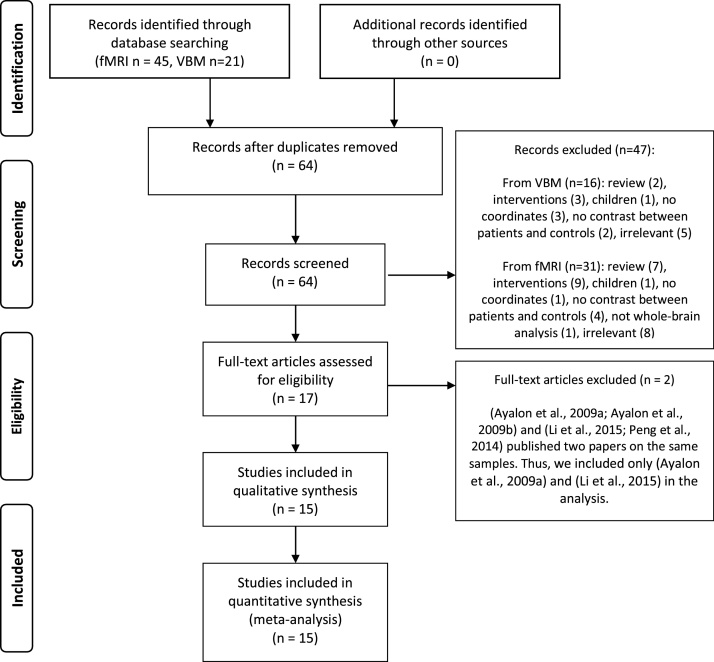
Paper selection strategy flow chart. fMRI = functional magnetic resonance imaging; VBM = voxel-based morphometry.

In accordance with the Preferred Reporting Items for Systematic Reviews and Meta-Analyses (PRISMA) statement (Moher et al., 2009), references for this meta-analysis were collected by a search of the PubMed database in April 2015, and by reference tracing of retrieved articles. Keywords for the search were as follows: (i) ((“Sleep Apnea Syndromes”[Mesh] OR “Sleep Apnea; Central”[Mesh] OR “Sleep Apnea; Obstructive”[Mesh]) OR sleep apnea) AND ((“functional magnetic resonance imaging”) OR “fMRI”); which resulted in 45 studies; (ii) (“Sleep Apnea Syndromes”[Mesh] OR “Sleep Apnea; Central”[Mesh] OR “Sleep Apnea; Obstructive”[Mesh]) OR sleep apnea AND ((“voxel-based morphometry”) OR “VBM”) that resulted in 21 studies (Fig. 1). No positron emission tomography (PET) studies met our criteria. Of note is that here; “study” reflects an individual scientific paper and “experiment” represents a single analysis or contrast of interest in a given study yielding localization information (i.e. OSA>controls; OSA<controls).

Only peer-reviewed cross-sectional studies that were published in English language and that compared groups of human adult OSA patients (above 18 years old) to healthy controls were included. The exclusion criteria were as follows:

• Case-reports, letter to editors, meta-analysis or review studies reporting no original data.
• Studies that did not report whole-brain analysis.
• Studies which did not report standard space coordinates.
• Studies that reported coordinates only in sub-sample.
• Intervention studies (pre/post treatment contrasts such as CPAP).
• Studies without a “control group” i.e. those focused only on a group of OSA patients.
• Studies where less than 7 patients were included in each group.

### 2.2 Data extraction

Two investigators independently extracted the information (M.T and A.A.S). Recorded data included the first author’s name, year of publication, age, gender and number of patients and controls, the imaging modality (resting-state/task fMRI or VBM) and type of task in the task fMRI studies. Moreover, we recorded the peak coordinates (x,y,z) in Talairach (Talairach and Tournoux, 1988) or Montreal Neurological Institute (MNI) (Evans et al., 1993) stereotactic space from all experiments and transformed all data into MNI coordinates for analysis (Lancaster et al., 2007). We used the extracted stereotactic coordinates to conduct the ALE meta-analysis.

If a publication did not report the coordinates of activation maxima, we contacted the authors. Two studies were excluded (Ayalon et al., 2009b, Peng et al., 2014) because the authors published two papers based on the same samples i.e. (Ayalon et al., 2009a, Ayalon et al., 2009b, Li et al., 2015, Peng et al., 2014). It is worthy to note that one study performed both VBM and rs-fMRI measurements (Zhang et al., 2013) and another one applied both VBM and task fMRI (Fatouleh et al., 2014) (Fig. 1).

### 2.3 Activation likelihood estimation

The statistical analyses were performed using the revised version of the activation likelihood estimation (ALE) (Eickhoff et al., 2012) based on coordinate-based meta-analyses (CBMA) (Eickhoff et al., 2009, Laird et al., 2009a, Turkeltaub et al., 2002). ALE assessed the significant convergence between activation foci from different experiments (e.g. OSA>controls, OSA<controls) for a given study in comparison with a random distribution of foci. More specifically, as the first step, ALE algorithm models the reported foci as center peaks of 3D Gaussian probability distributions that acknowledge the spatial uncertainty associated with each focus. The uncertainty is mainly due to between-subject variations (neuroanatomical variability and small sample sizes) and between-laboratory differences (various brain templates, normalization, and analysis strategies). The number of participants per experiment determines the width of the spatial uncertainty of any focus (Eickhoff et al., 2012). As the second step, the probability distributions of all activation foci in a particular experiment are combined for each voxel, which creates a modeled activation map (MA map). Thus, these MA maps summarize localization probabilities of studies and the final ALE map results from interpolation of these MA maps describing the convergence of results across all experiments. During the third step, an analytical approach based on a non-linear histogram integration is applied to test against the null hypothesis of randomly distributed foci and subsequently significant statistical threshold set at p < 0.05 family-wise error in cluster level (cFWE). The recent analysis approach tested for convergence by experiments (random effects) rather than foci (fixed effects) (Eickhoff et al., 2012); for further details and a summary of the ALE method please refer to (Eickhoff and Bzdok, 2013, Laird et al., 2009a, Laird et al., 2009b).

### 2.4 Functional decoding using the BrainMap database

To assess the functional roles of the abnormal brain regions in OSA, behavioral decoding using the BrainMap database was consequently performed. More specifically, we tested which types of tasks were more likely than chance to activate for each of the meta-analysis common regional gray matter loss and functional abnormalities (or seeds) (Laird et al., 2009a, Laird et al., 2009b, Rottschy et al., 2013). The behavioral domain and paradigm class meta-data categories from the BrainMap database were used for functional characterization of the clusters. At the time of analysis, the database included coordinates of reported activation foci and associated meta-data of more than 10,000 neuroimaging experiments (Laird et al., 2011, Laird et al., 2009b, Turner and Laird, 2012). Behavioral domains of the BrainMap database consist of several main categories including cognition, action, perception, emotion, and interoception, and their related sub-categories, which define the neural processes isolated by the respective contrast. However, paradigm classes specify the particular applied task (Fox et al., 2005) (see http://www.brainmap.org/scribe). For the functional characterization of common regional atrophy and functional abnormalities, all experiments in the BrainMap database that featured at least one focus of activation within the seed regions were determined based on reported activation coordinates. Subsequently, for each behavioral domain and paradigm class category, we identified regional functional profile by discerning taxonomic labels for which the probability of finding activation in the respective cluster is significantly higher than the overall chance across the entire BrainMap database. Significant level was identified as p < 0.05 using a binomial test (Caspers et al., 2014, Muller et al., 2013, Rottschy et al., 2013).

### 2.5 Whole-brain co-activation profiles

In order to map brain regions that feature significant co-activation with the regions identified in the OSA structural and functional meta-analysis, we performed meta-analytic co-activation modeling (MACM). More specifically, we tested how likely it is that the experiments activating the particular region also activate other brain voxels above chance (Eickhoff et al., 2011, Robinson et al., 2010). In order to perform MACM, first we identified all experiments in the BrainMap database that activate the convergent seeds. Then, quantitative meta-analysis was applied to test for convergence across the foci reported in the experiments. Inevitably, the highest convergence will be observed in the seed regions because experiments are already selected by activation in those seeds. Significant convergence of reported foci in other brain areas represents consistent co-activation or functional connectivity of other voxels with the seeds. More specifically, MACM provides information on the functional interactions of cortical modules based on their whole-brain co-activation pattern across the BrainMap database (Eickhoff et al., 2011, Laird et al., 2013).

## 3 Results

Fifteen publications that recruited 290 OSA patients and 290 healthy controls were included (Ayalon et al., 2009a, Ayalon et al., 2006, Fatouleh et al., 2014, Harper et al., 2003, Henderson et al., 2003, Joo et al., 2010, Li et al., 2015, Macey et al., 2006, Macey et al., 2003, Morrell et al., 2010, Prilipko et al., 2011, Santarnecchi et al., 2013, Torelli et al., 2011, Yaouhi et al., 2009, Zhang et al., 2013) (Table 1, Fig. 1). These publications collectively reported results from 30 experiments; of which 17 experiments were reported as the “OSA<controls” contrasts and 13 experiments for the “OSA>controls” contrasts.

### 3.1 Convergence of neuroimaging findings in OSA

**Fig. 2:**
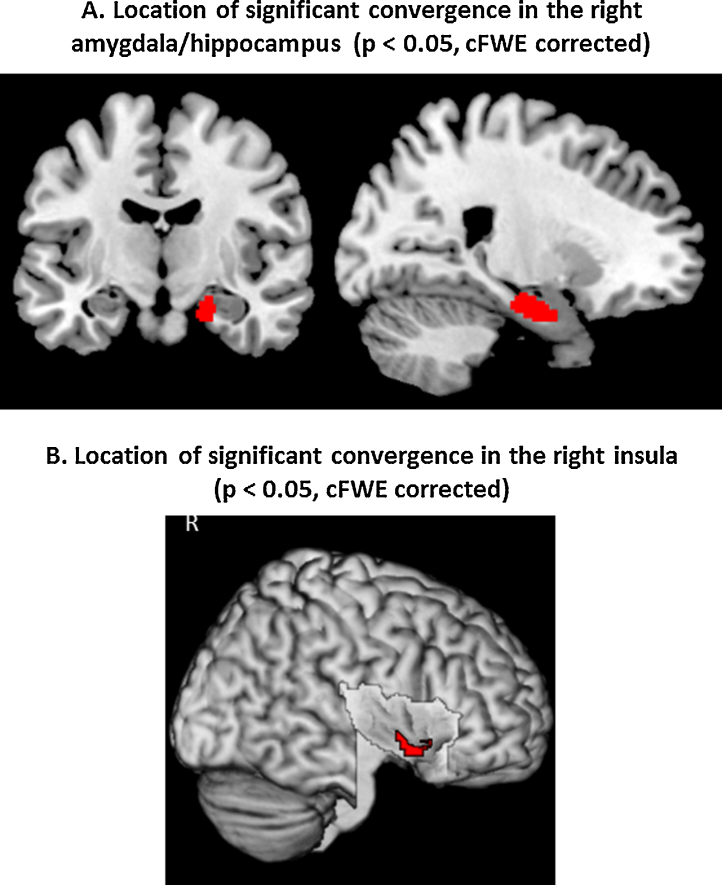
Convergence of structural and functional difference in obstructive sleep apnea compared with healthy controls. Location of the significant convergence of gray matter reduction and functional disturbance in the right basolateral nucleus of the human amygdala/hippocampus (A) and in the right central insula (B). Results are from the Activation Likelihood Estimation for sleep apnea meta-analyses. All activations are significant at P < 0.05 corrected for multiple comparisons using the family-wise error rate in cluster level (cFWE).

Testing for significant convergence across all eligible neuroimaging experiments comparing subjects with OSA to healthy controls yielded two significant clusters, one located in the right amygdala/hippocampus, the other in the right central insula (p < 0.05 FWE corrected in cluster level) (Fig. 2).

The cluster of convergence in the right amygdala/hippocampus was driven by a 62.5% contribution from task-fMRI studies (cognitive stimulation paradigms contributed 35% and sensory stimulation paradigms contributed 27.5%). Further 37.5% of contribution was driven by VBM studies. No contribution from resting-state fMRI was noted. The identified shared cluster of reduced grey matter volume and functional hypo-activation in OSA patients (local maximum: 22/-10/-22 in MNI space) was then anatomically allocated to the internal subdivision of the human right amygdala and hippocampus, as defined by histological (Amunts et al., 2005) and functional (Amft et al., 2015, Bzdok et al., 2013, Robinson et al., 2015, Robinson et al., 2010) criteria using the SPM Anatomy Toolbox (Eickhoff et al., 2005). Majority of the cluster volume (31%) appeared assigned to the basolateral nucleus of the amygdala, with smaller portions extending into the CA1 (14%), CA3 (13%), and subiculum (9%) (Fig. 2A). A second cluster of significant convergence was located in the right central insula (local maximum: 40/2/-4 in MNI space) (Cauda et al., 2012, Kurth et al., 2010). This result was almost completely driven by the hypo-activations in task-fMRI studies (99.9%). Among those, cognitive stimulation paradigms contributed 55.4% and sensory stimulation paradigms contributed 44.5% to the right central insula (Fig. 2B).

Supplementary analyses targeting convergence among specific aspects confirmed these key findings. We then tested for significant convergence across the functional MRI findings, pooling across task- and resting-state fMRI analyses in order to provide a global assessment of aberrant functional patterns in OSA patients. Accounting for multiple comparisons across the entire brain, a significant convergence was again identified in the right basolateral amygdala/hippocampus and right central insula. Subsequently, we sub-analysed only task-based fMRI studies, identifying the same regions as significant. Due to the low number of available experiments, resting-state functional imaging findings could not be reliable assessed in isolation at this point.

In summary, the performed series of quantitative meta-analyses on structural and functional neuroimaging findings in OSA patients revealed consistent evidence for primarily structural atrophy and functional disturbances (task-related hypo-activations) in the right amygdala/hippocampus and right central insula.

## 3.2 Functional decoding using the BrainMap database

**Fig. 3:**
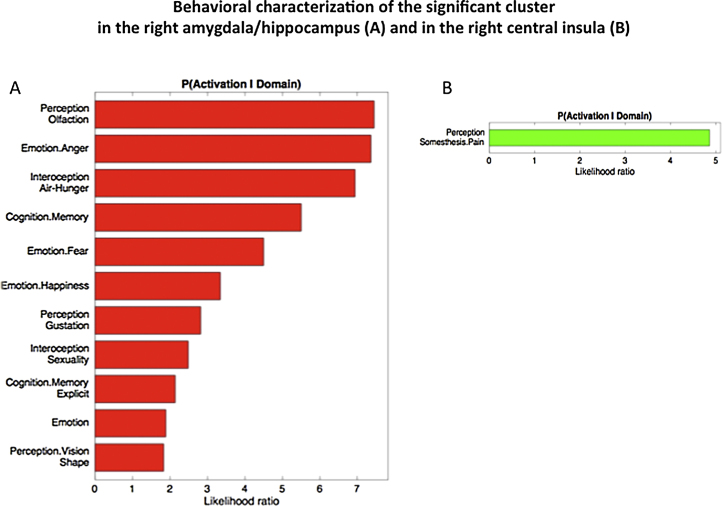
Behavioral characterization of the significant cluster in the right amygdala/hippocampus (A) and in the right central insula (B). Only behavioral domains significantly associated with the respective clusters at p < 0.05 (corrected for multiple comparisons) are illustrated.

In order to assess the functional roles of these two brain regions, which feature the most consistent evidence for OSA-related aberrations, we performed behavioral decoding using the BrainMap database. More precisely, we tested which types of tasks were more likely than by chance to activate the right basolateral amygdala/hippocampus and right central insula regions identified in the main ALE analysis. We found a significant (p < 0.05, corrected for multiple comparisons) association of the right basolateral amygdala/hippocampus region with affective and emotional processing, perception/interoception, memory-related processes, somatosensory functions (Fig. 3A). The cluster located on the right central insular cortex was reported as associated with somatosensory processing, and in particular with pain processing (p < 0.05 corrected for multiple comparisons) (Fig. 3B).

In summary, the behavioral decoding of the two identified regions via BrainMap functional database indicates their previous significant association with sensory and phylogenetic older limbic processes.

### 3.3 Whole-brain co-activation profiles

As our next step and in order to map brain regions that feature significant co-activation with the two identified regions, we performed MACM. In first instance, those experiments in BrainMap that feature activation in the region of the right basolateral amygdala/hippocampus and the right central insula were identified. Those regions that were more likely than by chance to be co-recruited with these seeds and may hence be considered as a part of the functionally connected networks, were then further investigated by an ALE meta-analysis across identified experiments.

**Fig. 4:**
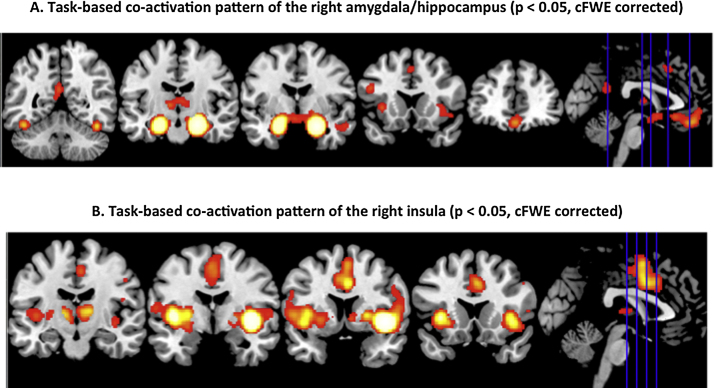
The results of meta-analytic connectivity modeling analysis. Task-based co-activation pattern of the right basolateral amygdala/hippocampus (A) and of the right central insula (B). All activations are significant at P < 0.05 corrected for multiple comparisons using the family-wise error rate in cluster level (cFWE).

The MACM analysis indicated significant (p < 0.05 FWE corrected in cluster level) co-activation of the right amygdala/hippocampus region with several bilateral brain regions. In particular, co-activations were found with the left amygdala/hippocampus (Bzdok et al., 2013, Robinson et al., 2015, Robinson et al., 2010), medial prefrontal (area FP2 (Bludau et al., 2014)), anterior cingulate cortex (areas s24 and s32 (Palomero-Gallagher et al., 2015)), posterior-medial frontal cortex (Nachev et al., 2008), and bilateral mid-fusiform gyrus (presumably corresponding to the fusiform face region) (Caspers et al., 2014). We also found significant co-activation with the bilateral thalamus (e.g. mediodorsal and anterior nuclei projecting to the prefrontal cortex and the ventral lateral and ventral anterior nuclei shown to project to motor and premotor cortices (Behrens et al., 2003)). In addition the anterior insula and adjacent ventro-lateral prefrontal cortex (vlPFC) (Kurth et al., 2010), as well as precuneus and posterior cingulate cortex (PCC) (Bzdok et al., 2015) were identified bilaterally (Fig. 4A).

Significant (p < 0.05 cFWE corrected) co-activation of the right central insula was largely symmetrically across both hemispheres. Co-activated regions comprised the bilateral parietal operculum (areas OP 1 and OP 4) (Eickhoff et al., 2006a, Eickhoff et al., 2006b) and adjacent inferior parietal cortex (areas PFop and PFcm (Caspers et al., 2008)), bilateral thalamus (Behrens et al., 2003) and posterior-medial frontal cortex (Bzdok et al., 2015). We also found significant co-activation with the bilateral ventral striatum and the basal forebrain. Finally, co-activated regions comprised the entire insula and perisylvian cortex including the adjacent ventro-lateral prefrontal cortex (Bludau et al., 2014, Cauda et al., 2012, Kurth et al., 2010) (Fig. 4B).

**Fig. 5:**
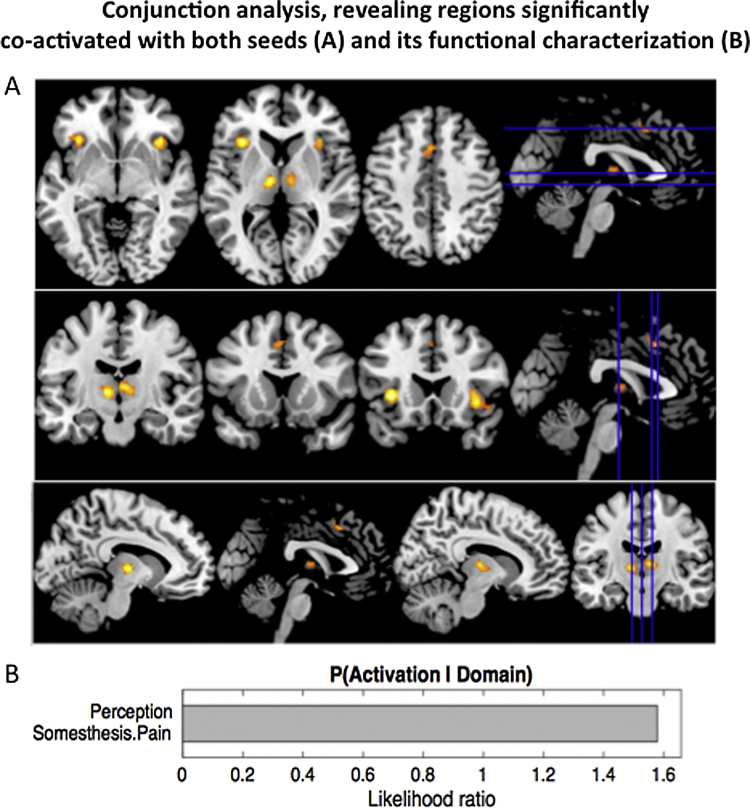
Conjunction analysis demonstrated regions significantly co-activated with both seeds (right basolateral amygdala/hippocampus and right central insula) (A). (B) Functional characterization of network shown in figure 5A (p < 0.05 corrected).

As the last step, we analysed the concurrence activations across both MACM analyses in order to identify regions that feature significant task-based co-activation with both seeds (right amygdala/hippocampus and right central insula). This analysis revealed the bilateral anterior insula and left opercular area OP 1, bilateral thalamus, as well as posterior-medial frontal cortex (Fig. 5A). Assessing the functions significantly associated with this entire network through behavioral characterization using the BrainMap database similarly suggested a strong connection with perception and somatosensory processing (Fig. 5B).

## 4 Discussion

It has been suggested that the diverse and often conflicting findings regarding brain structure and function in a variety of disorders may be ameliorated by a more finely tuned understanding of which structures in networks are most implicated (Celle et al., 2015, Eickhoff and Bzdok, 2013). In this vein, in our study, we have undertaken to gain greater understanding of core features of regional volume and activity alteration in patients with OSA across the published literature by using the ALE meta-analysis of currently available functional and structural imaging studies. This has been done with view to address the impact of diffuse changes in important areas on emotions, sensory and cognition impairments in patients with OSA.

In our study, the combined ALE quantitative analyses on group contrasts between patients with OSA and healthy controls highlighted structural atrophy and functional disturbances (task-related hypo-activations) in the clusters corresponding to the right basolateral amygdala/hippocampus and the right central insula (Fig. 2). These highlighted regions, the amygdala/hippocampus and the insular cortex, have so far been relatively neglected nodes in the OSA neurocircuitry fingerprint. The likely contribution of these structures to recognized deficits and disabilities in OSA is hence explored here further. Behavioral decoding analyses of these two regions demonstrated the possible dysfunction of emotional, sensory, and cognitive processes (Fig. 3). The results of MACM analysis inferred that the right basolateral amygdala/hippocampus and central insula also comprise a network with the bilateral anterior insula, posterior-medial frontal cortex and thalamus (Fig. 4, Fig. 5).

### 4.1 Amygdala/hippocampus ‘node’ and OSA

Convergent functional and structural alteration in the right basolateral amygdala and the CA1/CA3 regions of hippocampus and the subiculum (Fig. 2A) in our results appears to have been driven by contribution of both structural and functional neuroimaging.

#### 4.1.1 Basolateral nucleus of amygdala

It has been posited that one of the functions of the amygdala is to link sensory input to emotional responses that then guide behavior (Whalen and Phelps, 2009). Aberrant facial emotions processing, emotional blunting and even aberrant sexual behaviors, dysfunctional memory and olfactory processing (Davis and Whalen, 2001), have all been reported with damage to amygdala (Davis and Whalen, 2001, Paxinos, 2004). Apart from the emotional processing, it has also been shown that basolateral nucleus of amygdala is involved in spatial and motor learning (Davis and Whalen, 2001). The majority of listed deficits have also been reported to a smaller or larger degree in patients with OSA (Kryger et al., 2011, Rosenzweig et al., 2015).

The importance of amygdala as one of structures affected by the chronic process of OSA has been previously intimated by several studies (Fatouleh et al., 2014, Kheirandish-Gozal et al., 2014, Mukai et al., 2013). For instance, in children with OSA, during watching empathy-eliciting scenarios, the severity of OSA predicted less sensitivity to harm in the left amygdala (Kheirandish-Gozal et al., 2014). In general, a greater neural recruitment of regions implicated in cognitive control, conflict monitoring, and attentional allocation has been required in those children with OSA in order to perform at the same level as children without OSA (Kheirandish-Gozal et al., 2014). The results of our meta-analysis may go some way to explain an aberrant facial cues processing previously noted in children. Namely, our findings implicated the co-activation of nominally important regions and circuitry for this processing, e.g. the right amygdala region co-activation with the mid-fusiform gyrus, corresponding to the face region, and the anterior hippocampus, ventral striatum, contralateral amygdala, and the contralateral prefrontal cortex was found (Fig. 4A). Of all nuclei, the basolateral nucleus of amygdala appeared most affected according to the pooled results of the meta-analysis (Fig. 2A).

Preclinical investigations of the amygdala connectivity suggest that the basolateral amygdala receives sensory information from the thalamus, hippocampus and cortex and then activates or modulates synaptic transmission in target areas appropriate for the reinforcement signal with which the sensory information has been associated (Paxinos, 2004). In animals, amygdala has been shown as vital for learning procedures and stress-induced conditioning that involves pairings of potent and arbitrary chemosensory stimuli (Wang et al., 2006). For example, an animal study demonstrated that corticotropin-releasing factor receptors within the basolateral amygdala are involved in regulating fear-conditioned alterations in sleep (Wellman et al., 2013).

Similarly, and in keeping with these studies behavioral decoding of the regions highlighted by our meta-analysis suggested a significant association with affective and emotional processing, memory-related processes, and chemo-sensory processing (Fig. 3A).

Moreover, it has been suggested that the role of the amygdala in modulating momentary levels of vigilance in response to uncertainty underscores its likely importance for the etiology of anxiety disorders, MDD and PTSD (Davis and Whalen, 2001, Etkin et al., 2009, Tahmasian et al., 2013). The increased prevalence of these psychopathologies has been recognized in patients with OSA, possibly also suggesting a dual relationship. A meta-analysis of functional neuroimaging studies in PTSD and anxiety patients has suggested the basal portion of the amygdala as the major focus of hyperactivity (Etkin and Wager, 2007), the same part of amygdala suggested by findings of our meta-analysis (Fig. 2A).

Recent evidence further suggests that the amygdala is part of a complex network that mediates the formation of a larger repertoire of positive and negative emotions and it’s dysfunctions may lead to various psychiatric disorders (Langevin, 2012). The pivotal role for the basolateral amygdala in differentiation of stimuli, and subsequent prediction of either positive or negative outcomes, has been suggested (Namburi et al., 2015). Specifically, it has been shown that synaptic plasticity in the basolateral amygdala mediates the acquisition of associative memories of both end of emotional valences, and that different populations of neurons of that complex may encode fearful or rewarding associations (Namburi et al., 2015). For example, it appears that basolateral nucleus neurons that project to the nucleus accumbens undergo synaptic changes following reward conditioning, whilst those that project to centromedial nucleus of amygdala undergo synaptic changes following fear conditioning (Namburi et al., 2015). Hence, it follows that the importance of hypotrophy of such pivotal region such is basolateral nucleus of amygdala in patients with OSA should not be ignored. It is easy to postulate that this deficit must have reverberating impact on the patients’ ability to decipher the very valence of ongoing life experiences and that in others lead to emotional blunting such can be seen in Alzheimer’s disease (AD) or behavioural variant frontotemporal dementia (Rosen et al., 2002, Tahmasian et al., 2015b, Tahmasian et al., 2016).

Bzdok and colleagues highlighted the role of basolateral amygdala for processing and integrating environmental information and coordinating high-level sensory input, while the centromedial area is associated with mediating attentional, vegetative, and motor responses. Furthermore, they showed that the right basolateral amygdala is coactivated with the following regions: the left amygdala, dorsomedial prefrontal cortex, temporal pole, precuneus, inferior parietal cortex bilaterally, ventromedial prefrontal cortex, superior temporal gyrus/associative auditory cortex, middle frontal gyrus/frontal eye field, hippocampus, and posterior superior temporal sulcus on the left side (Bzdok et al., 2013). Our results are largely in agreement with the above-mentioned findings and also with the results of a previous study on amygdala that applied MACM (Robinson et al., 2010) (Fig. 4A). It also follows that dysfunction of emotional, sensory, and limbic processes reported in some patients with OSA might be partly explained with structural and functional alteration of right amygdala/hippocampus region (Fig. 3A).

#### 4.1.2 Hippocampus

The very possibility of a tripartite link between amygdala, memory impairment and OSA also posits itself as worthy of further exploration. The notion that activation of the amygdala during emotional arousal enhances memory in part by modulating plasticity in the hippocampus is not a new one (Bass and Manns, 2015, Kluver and Bucy, 1937, McGaugh et al., 2002, McIntyre et al., 2003, Pare, 2003). In addition, aberrant functional connectivity of amygdala and hippocampus may interact with dysfunctional intrinsic network activity in MDD (Tahmasian et al., 2013), which might be related to emotional memory disturbances in OSA as well. Moreover, an aberrant connectivity between the hippocampus and the cerebellum has been reported in OSA patients, with view that this may lead to alterations in a distributed memory system for associative learning (Rosenzweig et al., 2013a). In addition, bilateral enlargement of hippocampus in OSA patients has been reported previously (Rosenzweig et al., 2013b)

Of some note is that our results suggest higher damage to the anterior/ventral part of the hippocampal formation. In primates amygdala-projecting neurons are focally restricted to the most anterior (uncal) CA1 and pro-subiculum (Strange et al., 2014), and fMRI activations associated with emotional memory in humans have been found to be primarily in anterior regions (Strange et al., 2014). There is also evidence of double dissociation between semantic processing in the anterior hippocampus and non-semantic processing in the posterior hippocampus. One example of semantic processing that requires flexible expression of memory is transitive inference (Strange et al., 2014), or a form of inferential and deductive reasoning, another previously reported deficit in OSA patients (Lal et al., 2012).

Similar to our findings, Weng and colleagues found significant grey matter atrophy mainly on the right in the parahippocampus in their meta-analysis (Weng et al., 2014). Several preclinical studies also highlighted parahippocampus and hippocampal formation as important node contributing to the structural and functional abnormalities in OSA. For example, it has been demonstrated that the hippocampus is the region with high vulnerability to intermittent hypoxia, which may underlie the high frequency of neurobehavioral deficits observed frequently in OSA (Gozal et al., 2001b). Moreover, chronic episodic hypoxia during sleep impairs substantial region-selective neuronal loss within the CA1 region and leads to spatial learning impairments (Gozal et al., 2001a). The co-activation results of our study for the right hippocampal formation, (inclusive of anterior CA1 region), are also in agreement with the previously reported hippocampal co-activated networks (e.g. mPFC, bilateral superior frontal gyri, left hippocampus and parahippocampal gyrus, amygdala, cingulate cortex, thalamus, fusiform gyrus) and as such they might go some way towards explaining deficits in patients with OSA in perceptual, cognitive and affective processing domains (Robinson et al., 2015). In the same vein, the functional characterization of the regions highlighted by our study suggests deficits in the perceptual, cognitive and affective processing domains (Figs. 3 A, 4 A). Also, one can argue that recently shown association of OSA with AD and cognitive deficits noted in patients with OSA could be in part explained with hippocampal dysfunction, as previously demonstrated in AD (Osorio et al., 2015, Pasquini et al., 2015, Tahmasian et al., 2015b). In addition, another meta-analysis recently suggested that patients with AD could have 5 times higher rate of presenting OSA symptoms than healthy individuals (Emamian et al., 2016).

Of note is also that a majority of highlighted aberrant findings in our study were found to be non-dominant-sided. It has been suggested that there is a general hemispheric lateralization of perceptual processing, with lateralization of serial or local processing (on the left) versus parallel, global or holistic processing (on the right) (Igloi et al., 2010). In a similar manner, some authors argue that rather than providing a single common function, the two hippocampi provide complementary representations for navigation (e.g. places on the right and temporal sequences on the left), both of which likely contribute to different aspects of episodic memory (Igloi et al., 2010). The right hippocampus appears specifically involved in memory tasks requiring allocentric processing of spatial locations and hence its impairment may have clinical reverberations in patients’ accurate large-scale navigation (Igloi et al., 2010), and it may possibly also negatively affect the driving ability in OSA patients. By contrast, the left hippocampus appears to be involved in episodic/autobiographical memory (Igloi et al., 2010). Similarly, it has been suggested that during response monitoring, the right amygdala and rostral anterior cingulate cortex may mediate aversive conditioning to errors, whereas the left amygdala may underpin detrimental negative affect concerning performance (Polli et al., 2009). The possible clinical implication for our OSA patients would be that combined activity in these structures, which may serve to help us learn from our mistakes without becoming overly upset about them, is not optimal. Malfunction within this system could conceivably also contribute to the genesis of previously reported neuropsychiatric deficits prevalent in OSA, such as depression, emotional lability, anxiety or even aggravation of the PTSD.

Taken together, our study posits the amygdala and the hippocampal formation as important affected nodes in OSA neuropathology.

### 4.2 Insula and OSA

The right insular cortex has been highlighted as another cortical structure of importance by our results (Fig. 2B). So far, insular cortex has been somewhat ignored in theoretical constructs of likely neuropathology that underscores the affective and cognitive deficits in patients with OSA. Neuroanatomically, insula presents a nexus at the confluence of several neural pathways. It is densely interconnected with itself and with almost all cortical association regions (Cauda et al., 2012, Kelly et al., 2012, Kurth et al., 2010). It has been shown to receive sensory, somesthetic and interoceptive inputs from cortical areas and via the thalamus (Kelly et al., 2012, Uddin, 2015). It is also interconnected with the medial temporal lobe, amygdala, and basal ganglia (Kelly et al., 2012). It has been long proposed that the integration of all these inputs might present the neural substrate for human phenomenological experience, i.e. our own idiosyncratic conscious perception and understanding of a particular situation or phenomenon, further underscoring the importance of this structure (for detailed overview please refer to (Kelly et al., 2012).

Our data suggests that OSA patients have convergent hypo-activation of the right central insula compared to healthy controls (Fig. 2B). Novel insights into the functional organization and specialization of the insula suggest that activity in the insula correlates with the degree of subjective salience, whether it is influenced by homeostatic, emotional or cognitive factors (Kelly et al., 2012, Uddin, 2015). Moreover, the subdivisions of insular cortex have been shown to be co-dependent, and each subdivision participates to varying degrees in nearly every task domain that has been investigated, including those involving language, memory, sensory (from gustation and olfaction, to music perception), interoception, somesthesis and emotional processing (Uddin, 2015). Again, it can be argued that the majority of listed functions have been recognized as abnormal or lacking to a varied degree in some patients with OSA (Kryger et al., 2011).

The relative salience of the multitude of informational inputs during wakefulness determines those inputs which deserve attention (Uddin, 2015). An intrinsic brain system known as the ‘salience network’, with key nodes in the insular cortices, has a central role in the detection of behaviorally relevant stimuli and the coordination of neural resources (Uddin, 2015). Emerging evidence suggests that atypical engagement of specific subdivisions of the insula within the SN is a feature of many neuropsychiatric disorders, including of AD and MDD (Manoliu et al., 2013, Moon et al., 2014, Nagai et al., 2007). In OSA, localized cortical thinning has been reported in the region of insular cortices (Joo et al., 2013) and bilaterally anterior insular neuronal damage and increased glial activation has also been shown (Yadav et al., 2014). Similarly, selective functional disconnection between the right anterior insula and the medial prefrontal cortex was correlated with the severity of the OSA in a very recent study, whilst the functional disconnection between the insula and the posterior cingulate cortex was correlated with depressive scores and working memory performance of patients with OSA (Zhang et al., 2015). Of other sleep disorders, patients with insomnia have also been shown to have a greater involvement of the anterior insula, as well as insula BOLD correlation with EEG gamma frequency power during rest (Chen et al., 2014). Moreover, this increased involvement of the anterior/ventral insula was associated with negative affect. For instance, it has been suggested that here aberrant activation of the insula in arousal networks may underlie the misperception of sleep quality and subjective distress in insomnia (Chen et al., 2014). Given that insomnia and sleep apnea frequently co-exist (Luyster et al., 2010), it is tempting to postulate that recently highlighted divergent results of subjective versus objective complaints in OSA patients may also be a reflection of similar misperception and/or interoception of variety of bodily and cognitive functions in a subgroup of patients with OSA (Rosenzweig et al., 2015).

Cauda and colleagues demonstrated that anterior insula, mainly on the right side, plays an important role in saliency detection and cognition (Cauda et al., 2012). Moreover, the insula can be parcellated to the anterior part, which is characterized by an attentional pattern of connectivity with frontal, cingulate, parietal, cerebellar regions, whereas the posterior part is characterized by a more local connectivity pattern with connections to sensorimotor, temporal and posterior cingulate areas (Cauda et al., 2012). It has also been shown that posterior insula is mainly activated by interoception, perception and emotion (Cauda et al., 2012). In broad agreement with previous functional implications for insular cortices, the results of our meta-analyses implicate right central insular region as well as point to the deficits in perceptual, somatosensory and affective processing (Figs. 3 B, 4 B). Arguably our findings also suggest that sensory and cognitive task-related modulations of the altered wider neurocircuitry in sleep apnea patients lead to a weaker central insular cortex’s functional connectivity and activation during a given task, by comparison to healthy volunteers. However, the possibility that, as of yet unclear maladaptive structural process in sleep apnea patients, leads to plastic alterations and to the “functional” deactivation of this very region, should not be overlooked.

In that vein and regarding hemispheric lateralization of insular changes highlighted by our study, an interesting study suggests insight into the potential mechanisms behind the pathological process at hand (Yadav et al., 2014). Namely, Yadav and colleagues recently showed bilateral insular neuronal damage in OSA patients with higher glial activation and neuroinflammation on the left (Yadav et al., 2014). The authors speculated that asymmetrical outcome could stem from the larger cerebral blood flow on the right side with the consequences of hypoxemic periods during apnea having a relatively reduced effect on the right over the left side (Yadav et al., 2014). In view of our results, however, another interesting possibility presents itself. In contrast to their finding, our results suggest higher impact on the right and conceivably may also suggest an adaptive role for the microglial activation early on, or at certain stages, during the chronic process of OSA in some patients. How, when and which of the neuroinflammation processes may be protective/adaptive, and which detrimental/maladaptive, has become one of the crucial questions in fields of neurodegenerative, psychiatric, traumatic and ischemic diseases, and it is of further interest that many of diseases (etc. MDD, AD, stroke) have been found co-morbid or even prevalent in patients with OSA (Rosenzweig et al., 2015).

### 4.3 The convergent role for amygdala and insula?

In order to perform a particular neural function, to form a thought or an emotion, a set of brain networks or systems needs to transiently interact (Sala-Llonch et al., 2012). The function and structure of such networks is of particular interest given that abnormalities in the interactions of network components can play a critical role in neuropsychiatric disorders, with damage to specific functional connectivity nodes and networks giving rise to distinct neurological and psychiatric syndromes (Bassett and Bullmore, 2009, Seeley et al., 2009, Tahmasian et al., 2016, Tahmasian et al., 2015a, Tahmasian et al., 2015b, Tahmasian et al., 2013). The results of our conjunctional meta-analysis point to functionally important co-activation between our seeds (in the right amygdala/hippocampus and right central insula) and the bilateral anterior insula, bilateral thalamus, as well as the posterior-medial frontal cortex (Fig. 5A). Assessing the behavioral characterization of both seeds using the BrainMap database demonstrated significant involvement of somatosensory and affective processing (Fig. 5 B). These findings are in agreement with previous MACM and behavioral decoding studies on the right amygdala/hippocampus and right insula (Bzdok et al., 2013, Cauda et al., 2012, Kurth et al., 2010, Robinson et al., 2015, Robinson et al., 2010).

The regions highlighted by our meta-analyses: the right amygdala/hippocampus and right central insula all exist, or contribute to, the flow of information as connection hubs on one of the three important intrinsic connectivity networks: the default mode network (DMN), the central executive network (CEN) and the salience network (SN). Of these, CEN (including the dorsolateral prefrontal cortex and posterior parietal cortex) and DMN (including the medial prefrontal cortex (mPFC)) and PCC are two important anti-correlated cognitive-related networks (Menon, 2011, Sala-Llonch et al., 2012). The DMN plays a competitive role with the majority of task-related networks and the activation of a cognitive task-related network is commonly accompanied by deactivation of the DMN (Buckner et al., 2008, Sala-Llonch et al., 2012). It has been shown that the connectivity within DMN regions in task-fMRI studies contributes to the facilitation or monitoring of cognitive performance, and that the differences in functional coupling within DMN regions can predict differences in cognitive performance (Sala-Llonch et al., 2012).

Over the years, numerous neuroimaging studies have been performed to identify structural and functional brain impairments in OSA patients in order to explain for noted emotional and cognitive deficits, including altered brain activation and deactivation in the CEN, DMN and SN (Zhang et al., 2015, Zhang et al., 2013). Defective deactivation of DMN in patients with OSA during task has been suggested by findings of one study, which also proposed the intermittent hypoxia damage as a more likely culprit behind observed aberrant connectivity (Prilipko et al., 2011). In addition, differential compensatory spatial recruitment of the task positive network and DMN has been demonstrated, with a different pattern of spatial recruitment and deactivation noted in comparison to healthy controls (Prilipko et al., 2011). An altered activation of the CEN and deactivation of the DMN during working memory tasks in OSA patients has also been shown (Zhang et al., 2015). Similarly, in rs-fMRI studies functional disconnection in brain areas of the CEN and DMN in OSA patients has been reported (Zhang et al., 2013). Taken together, these findings might be taken to suggest a role for the functional impairment of the CEN and DMN in cognitive deficits in patients with OSA. Of note, some of those alterations have been reported as reversible and CPAP treatment has been shown to increase the connectivity of the DMN in elderly patients with OSA and to attenuate cortical thinning (Dalmases et al., 2015).

The functional disconnection of the insular cortex with the CEN and DMN networks has been reported in normal aging and many neuropsychiatric, neurodegenerative disorders (Tahmasian et al., 2013, Tahmasian et al., 2016, Manoliu et al., 2013). In keeping with the findings of our meta-analysis that highlighted ventral insular cortex as an important node in somatosensory, neurocognitive, perceptual and affective deficits in patients with OSA, a recent study has demonstrated decreased functional connectivity of the right insular cortex with the main nodes of the DMN. This has been taken to indicate the functional disconnection between the SN and DMN in OSA patients (Zhang et al., 2015). In addition, the decrease in functional connectivity between the right AI and the mPFC has been significantly correlated with the apnea and hypopnea index (AHI value). This correlation has been taken to suggest the injury by the intermittent hypoxia as the most likely culprit underscoring the aberrant disconnection (Zhang et al., 2015). It has been suggested that the functional disconnection between the insular cortex and the DMN may in itself be sufficient to lead to aberrant cognitive control signals and to result in cognitive impairment in OSA patients (Zhang et al., 2015). In addition, in another study, the abnormal insular cortex metabolites in adults with OSA showed significant correlations with disease severity and neuropsychological status, suggesting not just cognitive but also emotional/affective impact of this disconnection (Yadav et al., 2014).

Finally, we found significant connectivity between the seeds (the right amygdala/hippocampus and right central insula) and bilateral anterior insula and left opercular area, bilateral thalamus, as well as posterior-medial frontal cortex (Fig. 5A). This pattern closely resembles the canonical frontoparietal executive control network (e.g. CEN) identified in many studies of cognitive control over emotional and non-emotional material (Etkin et al., 2009). This coordinated network has also been observed using rs-fMRI and as previously noted; it has been reliably dissociated from the SN (Etkin et al., 2009). The executive control network in healthy controls does not normally include the amygdala or insula, and thus coupling of these two structures with this network and impaired dissociation with SN, and possibly also impaired deactivation of DMN in patients, likely further reflects a chronic disorder driven network-level neural adaptation.

In summary, the noted aberrant connectivity could be argued to underlie previously reported dysmetric deficits of affect, attention, information processing and visuo-motor control (Rosenzweig et al., 2015, Rosenzweig et al., 2014). Of further note, similar aberrant connectivity with some of these networks has also been recently reported in patients with generalised anxiety disorder, MDD, and PTSD (Brown et al., 2014, Etkin et al., 2009, Manoliu et al., 2013, Tahmasian et al., 2013).

### 4.4 Research in context

Recently, an ALE meta-analysis on eight VBM studies found significant reductions in gray matter of the bilateral parahippocampal (more robust on the right side) and frontotemporal regions (less-pronounced) in patients with OSA (Weng et al., 2014). Similarly, our meta-analysis suggests convergent grey matter atrophy and functional hypo-activation in the basolateral amygdala and the hippocampal formation. Unlike their study, our ALE analysis included both structural and functional (e.g. task-fMRI and rs-fMRI) neuroimaging studies in order to comprehensively assess both abnormalities in OSA. Due to our stringent inclusion criteria we have excluded several structural studies that were otherwise incorporated by them (Weng et al., 2014), For example, we excluded all those previous studies that did not report standard space coordinates (Macey et al., 2002, Morrell and Twigg, 2006), studies that did not find group difference between OSA patients and controls (O'Donoghue et al., 2005), and one interventional study (Canessa et al., 2011). In addition, in our meta-analysis we used as a statistically significant cut off point P < 0.05 corrected for multiple comparisons using the FWE in cluster level. This cut off point is more conservative than False Discovery Rate (FDR) used in that meta-analyses (Weng et al., 2014) and as such has likely had an impact on our results. Moreover, we indentified behavioral characterization of the seed regions using the BrainMap database and also task-based co-activation patterns of functionally connected areas to these regions, which have not been previously done.

### 4.5 Potential limitations of the present study

Coherent summary of the neuroimaging literature about the impact of OSA on the brain that is growing in scope and complexity requires increasingly sophisticated tools for synthesizing findings across studies. The meta-analysis has been accepted as an important tool to develop new hypotheses on structure–function correspondence and to establish consensus on the locations of functional regions in diseases such as OSA across previously published studies (Eickhoff and Bzdok, 2013, Eickhoff et al., 2012, Wager et al., 2009). Nonetheless, the meta-analysis itself is not fully immune of limitations that arise from characteristics of the primary studies under review (Wager et al., 2009). It is hence of importance to recognize that despite best efforts to circumvent many limitations connected to single studies and the stringent exclusion and inclusion criteria used, the studies included in our meta-analysis differed regarding design, methodology, and the study population. Unfortunately, none of the above mentioned analysis, controlled for age or gender covariates across studies due to current methodological limitations. In addition, the majority of the included studies in our meta-analysis investigated the brain activation or changes in a sample of men without consideration of possible gender differences and thus, we were not able to identify the activation patterns separately for both genders. Similarly, it is impossible to account for separate activation patterns at various stages of OSA in different study populations, and to discern the temporal vector of the noted changes. Additionally, it should be also noted that although we included all available neuroimaging studies that satisfied our predetermined criteria, it is still possible that the sample size for our meta-analysis has underpowered our findings. For example, in this study our exclusion criteria stipulated exclusion of interventional studies and it is possible that their inclusion might have strengthened our findings. Future studies can apply various cognitive and emotional task fMRI to understand different angels of the associated neuropsychiatric symptoms of OSA (e.g. memory loss, depression). Moreover, we did not find any results from rs-fMRI experiments maybe because of low number of available rs-fMRI studies. rs-fMRI as a promising non-invasive tool is, however, currently widely employed to measure functional connectivity alteration in different neuropsychiatric disorders and maybe future studies can implement it to assess the intrinsic functional abnormalities in OSA.

Beside those primary source-related issues, the very determination of the consistent brain structures differences remains the problem in various meta-analysis methods, including in the ALE method, which was utilized in this study. The CBMA method uses precise coordinates (rather than general regional labels) as its input. The issue arises from the fact that these peak coordinate foci are limited indicators of the location of a significant anatomical difference (Fox et al., 2014, Wager et al., 2009). Finally, during the ALE analysis, overlapping clusters of difference are commonly found by averaging across different peak coordinates, increasing the risk that with two or more relatively nearby peak foci, ALE will find an average, ‘significant’ cluster somewhere between these foci in a brain region which was actually not reported in the source studies (Fox et al., 2014). On the other hand, one study recommended using image-based rather than CBMA method in ALE meta-analysis, which was applied in this study. The reason is the CBMA method provide less information from each study (Salimi-Khorshidi et al., 2009). However, the original data is often very difficult to obtain from previous studies than reported peak coordinates. Hence, CBMA is still the standard ALE approach to detect convergent regional abnormalities in neuropsychiatric disorders (Eickhoff and Bzdok, 2013).

## 5 Conclusions

The impact of oxidative and neuroinflammatory effects of OSA on the right amygdala/hippocampus along with the right insular cortex and other subcortical and cortical structures plays an important role, which has been previously suggested to underscore several of subjective and objective cognitive and emotional complaints of adult OSA patients (Rosenzweig et al., 2015). Here, we present findings of the meta-analysis on neuroimaging studies that also implicates the right amygdala/hippocampus complex and the insular cortex as important nodes on the affected cognitive and affective circuitry in OSA patients. Moreover, in accordance with this and previous studies, the behavioral characterization of the entire highlighted network using the BrainMap database suggested implications for the emotional and memory related functions, as well as somatosensory processing in the affected patients. A MACM analysis demonstrated that the right amygdala/hippocampus and insula are part of a joint network comprising the anterior insula, posterior-medial frontal cortex and thalamus. Further, our study strongly suggested non-dominant lateralization of noted chronic deficits in OSA. It was outside the scope of this paper to provide any detailed mechanistic insights behind this phenomenon, however, its significance should not be ignored and should be further explored in future studies.

Taken collectively, neuroimaging and neurophysiological studies in patients with OSA have delineated a putative regional “fingerprint” of OSA-induced brain injury. They purport a disconnection of the fronto-parietal regions and a disruption of the thalamocortical oscillator, with involvement of the hippocampal formation (Rosenzweig et al., 2016a, Rosenzweig et al., 2016b).

One of the challenges for future research will be to establish and differentiate the nuanced task fMRI profiles and patterns of functional connectivity of particular subdivisions of the insula and amygdala with the intrinsic brain networks in OSA patients. Given the burgeoning body of research into aberrant connectivity of intrinsic brain networks and their implication in disorders such as AD and other neuropsychiatric and neurodegenerative disorders, the ability to decipher correct or convergent biomarkers for each of these disorders can not be overstated. Ideally, any such research would also make it possible to delineate specific contribution of several of neuropathological facets of OSA injury on any changes noted, e.g. sleep fragmentation versus the impact of intermittent hypoxia versus any other confounding factors such as obesity. This has so far been difficult to implement, but future careful experimental designs might help with this issue. In particular targeted cognitive and emotional tasks fMRI studies could be well positioned to explore some of the previously reported neurocognitive and neuropsychiatric symptoms associated with OSA. Finally, it is hoped that the findings presented here may offer a tentative first step towards this task, as well as to provide an initial theoretical framework for interpreting the aberrant activity within these network nodes.

## Conflict of interest

The authors declare that the research was conducted in the absence of any commercial or financial relationships that could be construed as a potential conflict of interest.

## Figures and Tables

**Table 1:** Studies entered into the meta-analysis are listed based on the year of publication and further alphabetically for each year. OSA = Obstructive sleep apnea; VBM = Voxel-based morphometry; fMRI = Functional magnetic resonance imaging; rs-fMRI = Resting-state fMRI; BMI = Body Mass Index. * Age was matched between groups and the authors reported only one value for two groups; ** Standard errors or confident intervals have been transformed to standard deviations (SD).

	Author, year	Number of subjects (OSA/controls)	Number of male subjects (OSA/controls)	Age of OSA patients/controls (Mean ± SD)	Imaging modality	Number of foci	Covariates
1	Li et al., 2015	25/25	25/25	39.4 ± 1.7/39.5 ± 1.6	rs-fMRI	2	Age, years of education
2	Fatouleh et al., 2014	17/15	15/12	55 ± 12.4**/53 ± 11.6**	fMRI, VBM	25	Age, sex, total brain volume
3	Santarnecchi et al., 2013	19/19	16/14	43.2 ± 8/41 ± 6	rs-fMRI	16	Age, gender, total brain volume and BMI
4	Zhang et al., 2013	24/21	24/21	44.6 ± 7.4/40.6 ± 11.4	rs-fMRI, VBM	7	Age
5	Prilipko et al., 2011	17/7	17/7	43.2 ± 8.4*/43.2 ± 8.4*	fMRI	25	Nocturnal desaturation time and BMI
6	Torelli et al., 2011	16/14	13/9	55.8 ± 6.7/57.6 ± 5.2	VBM	2	Demographic characteristics, comorbidities and intracranial volume, education
7	Joo et al., 2010	36/31	36/31	44.7 ± 6.7/44.8 ± 5.4	VBM	27	Age, intracranial volume
8	Morrell et al., 2010	60/60	57/55	47.3 ± 12.1**/46.1 ± 11.5**	VBM	2	Age, sex and intracranial volume
9	Ayalon et al., 2009a	14/14	13/13	45.6 ± 11.7/43.6 ± 8.6	fMRI	10	Age
10	Yaouhi et al., 2009	16/14	15/13	54.75 ± 5.71/52.71 ± 7.01	VBM	7	Age
11	Ayalon et al., 2006	12/12	11/11	44.2 ± 11.9/43 ± 9.1	fMRI	23	–
12	Macey et al., 2006	7/11	7/11	46 ± 13.2**/47 ± 9.9**	fMRI	37	–
13	Harper et al., 2003	10/16	10/16	46 ± 12/47 ± 10	fMRI	19	–
14	Henderson et al., 2003	8/15	8/15	44 ± 11.3**/45 ± 11.6**	fMRI	12	–
15	Macey et al., 2003	9/16	9/16	45 ± 12/47 ± 10	fMRI	12	–
